# ESUR/ESUI position paper: developing artificial intelligence for precision diagnosis of prostate cancer using magnetic resonance imaging

**DOI:** 10.1007/s00330-021-08021-6

**Published:** 2021-05-15

**Authors:** Tobias Penzkofer, Anwar R. Padhani, Baris Turkbey, Masoom A. Haider, Henkjan Huisman, Jochen Walz, Georg Salomon, Ivo G. Schoots, Jonathan Richenberg, Geert Villeirs, Valeria Panebianco, Olivier Rouviere, Vibeke Berg Logager, Jelle Barentsz

**Affiliations:** 1 Department of Radiology, Charité University Hospital, Augustenburger Platz 1, 13354 Berlin, Germany; 2 Berlin Institute of Health, Berlin, Germany; 3 Paul Strickland Scanner Centre, Mount Vernon Cancer Centre, Northwood, UK; 4 Molecular Imaging Branch, National Cancer Institute, NIH, Bethesda, MD USA; 5 Joint Department of Medical Imaging, Sinai Health System, University Health Network, University of Toronto, Toronto, Canada; 6 Department of Radiology and Nuclear Medicine, Radboud University Medical Center, Nijmegen, The Netherlands; 7 Department of Urology, Institut Paoli-Calmettes Cancer Centre, Marseille, France; 8 Martini-Klinik am UKE, University Hospital Hamburg, Hamburg, Germany; 9 Radiology & Nuclear Medicine, Erasmus MC, Rotterdam, The Netherlands; 10 Department of Radiology, Netherlands Cancer Institute, Amsterdam, The Netherlands; 11 Department of Imaging, BSUH NHS Trust, Brighton, UK; 12 Department of Radiology and Nuclear Medicine, Ghent University Hospital, Ghent, Belgium; 13 Department of Radiological Sciences, Oncology and Pathology, Sapienza/Policlinico Umberto I, Rome, Italy; 14 Department of Urinary and Vascular Imaging, Hospices Civils de Lyon, Lyon, France; 15 Faculté de médecine Lyon-Est, Université de Lyon, Université Lyon 1, Lyon, France; 16 Radiological Department, Copenhagen University Hospital in Herlev-Gentofte, Herlev, Denmark

**Keywords:** Artificial intelligence, Deep learning, Prostate cancer, Multiparametric magnetic resonance imaging, Image-guided biopsy

## Abstract

**Abstract:**

Artificial intelligence developments are essential to the successful deployment of community-wide, MRI-driven prostate cancer diagnosis. AI systems should ensure that the main benefits of biopsy avoidance are delivered while maintaining consistent high specificities, at a range of disease prevalences. Since all current artificial intelligence / computer-aided detection systems for prostate cancer detection are experimental, multiple developmental efforts are still needed to bring the vision to fruition. Initial work needs to focus on developing systems as diagnostic supporting aids so their results can be integrated into the radiologists’ workflow including gland and target outlining tasks for fusion biopsies. Developing AI systems as clinical decision-making tools will require greater efforts. The latter encompass larger multicentric, multivendor datasets where the different needs of patients stratified by diagnostic settings, disease prevalence, patient preference, and clinical setting are considered. AI-based, robust, standard operating procedures will increase the confidence of patients and payers, thus enabling the wider adoption of the MRI-directed approach for prostate cancer diagnosis.

**Key Points:**

*• AI systems need to ensure that the benefits of biopsy avoidance are delivered with consistent high specificities, at a range of disease prevalence.*

*• Initial work has focused on developing systems as diagnostic supporting aids for outlining tasks, so they can be integrated into the radiologists’ workflow to support MRI-directed biopsies.*

*• Decision support tools require a larger body of work including multicentric, multivendor studies where the clinical needs, disease prevalence, patient preferences, and clinical setting are additionally defined.*

## Introduction

In recent years, high-level evidence has emerged to support the use of MRI for prostate cancer detection in both biopsy-naïve patients, and for men at persistent suspicion despite negative results on prior biopsies. In the Oxford Cochrane review [1], the MRI pathway (MRI ± MRI-directed biopsy) was found to have more favourable diagnostic properties compared to systematic biopsy approaches.

The MRI-directed pathway has been accepted by multiple national and international guidelines for prostate cancer diagnoses. The Prostate Imaging Reporting and Data System (PI-RADS) standard for multiparametric MRI (mpMRI) evaluation and reporting [2] has also been adopted into multiple guidelines to action the use of biopsies [3–5]. As a result, there is a worldwide increase in the demand for MRI for diagnosis and MRI-influenced guided biopsies.

The major benefits of the MRI-pathway in biopsy-naive men are reductions in the number of men needing biopsies, reducing the number of biopsy cores used to make diagnoses [6], and in decreased detection rates of indolent cancers. In patients with negative results from prior biopsies, increased diagnoses of clinically significant cancer is an added benefit [7]. Additionally, there is accumulating data from randomized studies showing improved diagnoses of clinically significant cancer in biopsy-naïve men [8, 9]. Improved diagnoses of clinically significant cancer in men with positive MRI results are subject to higher variability, with high false-positive rates [10].

The delivery of diagnostic benefits is very dependent on high reader expertise [11]. Expert readers with good images make more accurate diagnoses with less uncertainty. High levels of expertise also enable the adoption of MRI approaches that avoid contrast medium injections (biparametric MRI; bpMRI) [12–14], which can help to increase patient throughput at a lower cost. High reader expertise also minimizes variations [15] in clinically significant cancer yields within the MRI suspicion categories, thus improving the uniformity and reliability of MRI findings for clinical decision-making. Also, expert readers have a lower percentage of uncertain diagnoses. However, there is a recognized steep learning curve for radiologists in prostate MRI interpretations also.

There are growing calls for high end-to-end quality of the MRI-directed diagnostic chain, through the effective working of the multidisciplinary teams. Delivering high-quality patient care by utilizing the MRI-directed pathway is challenging due to increasing demands on radiologists’ time and a workforce that is not fully trained to interpret prostate MRI to a high standard. High-quality delineation of target lesions is essential to guide MRI-directed biopsies and for subsequent therapy planning. However, both the detection of biopsy targets and the delineations needed in preparation for biopsy are time-consuming tasks, which are not reimbursed in many practices.

In order to deliver the benefits of the MRI-pathway, there is an important need to work efficiently while minimizing variations in MRI data acquisitions, image quality, image interpretations, and to decrease the number of diagnostic steps needed to identify men who are likely or unlikely to have clinically significant cancers [7]. There is also a corresponding need to control the quality of MRI-directed biopsy procedures by identifying the most suspicious areas to sample [16, 17]. Artificial intelligence approaches have the potential to increase work efficiency and minimize the variability of the results obtained.

Artificial intelligence (AI) systems can potentially be helpful by automating multiple steps in the MRI pathway [18–20], not only by alleviating the aforementioned demands on radiologic tasks but also by diminishing variability in diagnostic performance. Several groups have set out to explore AI technologies for the diagnosis of prostate cancer on MRI, but thus far, there has been limited adoption into clinical practice. The reasons for this are multiple and include the fact that most AI systems are at a very early stage of development in single-centre settings, with limited user interfaces, lack integration into the clinical workflow, and importantly have not been validated for clinical use. Recently, an AI-based system gained CE-compliance in the European Union as a medical device, indicating a positive development [21–23].

Two recent articles analyzed the state-of-the-art and quality of radiomics and AI-based approaches (deep and non-deep learning) in prostate MRI [24, 25]. The studies found that while the results are promising in terms of reported performance, with good accuracy, there was a need for broader comparability for systems against a common gold standard, and improved quality in the range of studies conducted that controlled biases with systematic validation strategies before broader clinical adoption could be undertaken.

Thus, multiple important steps for the establishment of AI in prostate MRI diagnosis have not yet been taken. This whitepaper aims to outline the necessary prerequisites and building blocks for the successful implementation of a clinically relevant AI for the diagnostic use of prostate MRI, reflecting the views of the Prostate MRI Working Group of the European Society of Urogenital Radiology (ESUR), European Association of Urology (EAU) Section of Urological Imaging (ESUI).

## Characteristics of artificial intelligence systems for prostate MRI in cancer diagnosis

### Definition of terms

Artificial intelligence (AI) is a broad field encompassing several technologies which deduce (“learn”) decision-making rules (“model”) directly from representative data (“dataset”) to achieve prespecified goals [26, 27]. That is, most AI systems create a “model” based on a training dataset which is then used to “infer” the properties of never-seen data for a specific purpose. The dataset used is divided into different parts during the process: beforehand, a representative part of the data is set away to later determine the performance of the model (“test dataset”). The remaining data is used to create the model (“training dataset”). For some methods, especially those with iterative rounds of parameter optimization, it is further necessary to split the training data: one dataset to deduce the model parameters (again referred to as “training dataset”) while another part is used to determine improvements between the iterations of the model creation process (“validation dataset”). It is important to note that the split between the training and validation dataset can be dynamic during the training, while the test dataset should always be separate from the training data and only be used to ascertain the performance of the model.

**Fig. 1:**
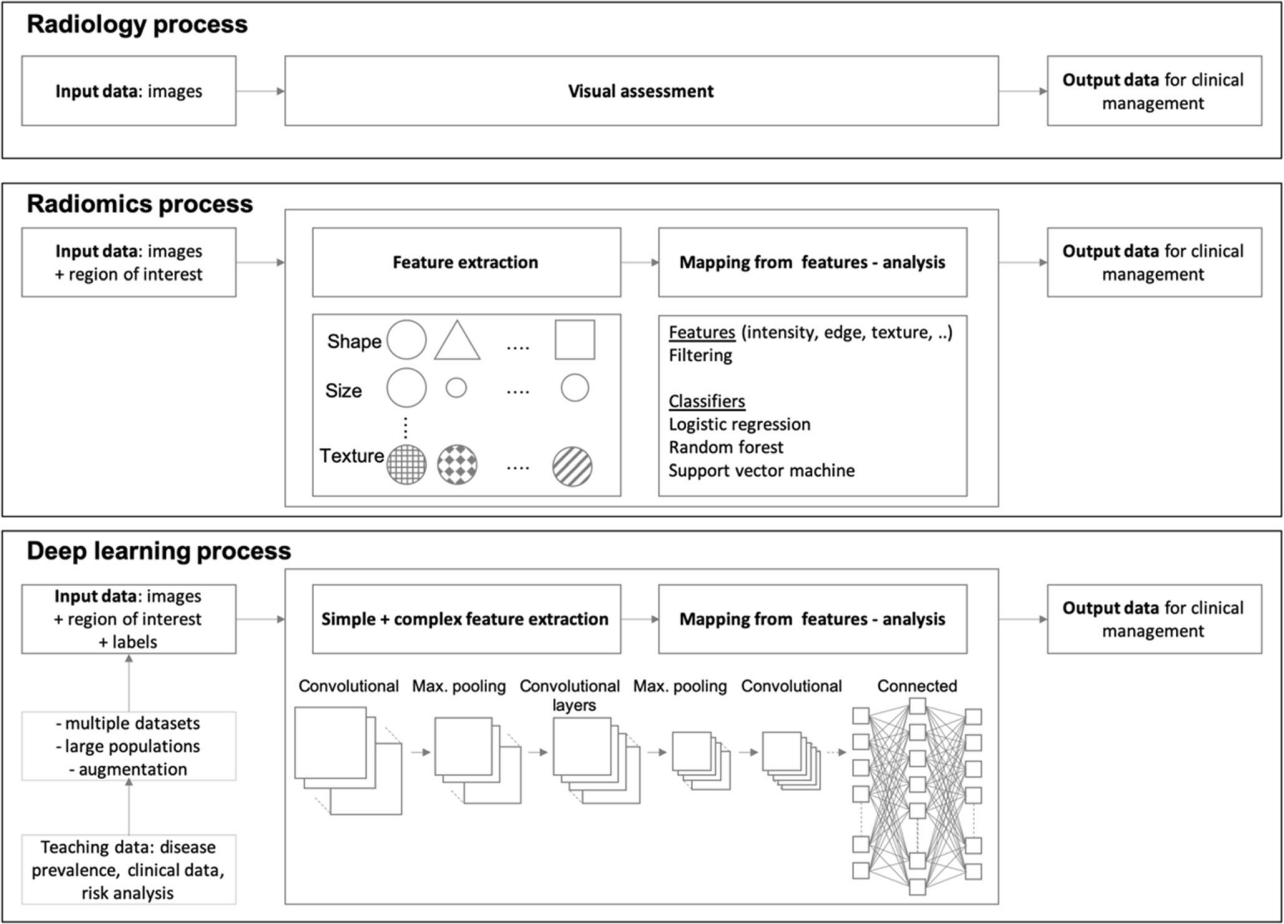
Comparative workflows for classic radiology, radiomics, and deep learning approaches to medical diagnosis: Workflows for the “classic” radiology process (top), the radiomics approach (middle), and the deep learning process (bottom). Only a few quantitative features are used in the radiological assessments, which are mostly based on subjective, visually assessed features incorporating few quantitative measurements such as size, ADC value, Hounsfield unit (HU), or relaxation rates/times. Radiomics, in contrast, systematically assesses a broad set of predefined features (e.g. shape, size, first-order texture features) which can additionally be filtered and searched for, to define patterns relevant to pathology using statistical methods. Deep learning (and other AI-based techniques) do not rely on predefined features but instead create independent features (where complex features are a composition of simpler ones) within artificial neural networks to distinguish between the desired target classes. All methods of analysis ultimately aim to guide clinical management of future patients with similar characteristics to the learning and validation datasets

This learning process can be supervised, semi-supervised, or unsupervised. The process is supervised when the outcome or class of each case is available during the training process. The process is unsupervised when the predictions are structured autonomously, and classes are derived from the input data. AI systems are hereby classified through the structure of the used models: Classic machine learning models employ non-neural network techniques like support vector machines (SVM), random forests, linear discriminant analysis (LDA), naïve Bayes classifiers, or k-nearest neighbour or neural networks with models with few or no layers (shallow neural networks); deep learning refers to models with many concatenated layers of artificial neurons to achieve the classification goal. In many instances, the feature extraction process will be separated for classic machine learning and integrated for deep learning, although this can vary from implementation to implementation (Fig. 1).

Many methods are used for model creation including artificial neural networks, statistical or logical analyses, and frequent combinations of methods are used [28].

AI for image analysis has recently experienced several technological advances fuelled by the growth in computing power, availability of annotated digital imaging datasets, and the development of new algorithms. Most AI applications for radiology apply some form of convolutional neural network which belongs to the family of “deep learning” techniques [29]. These networks can create models for segmentation, detection, or classification within the imaging data. When other patients’ relevant meta-data (e.g. laboratory values or text reports) are incorporated into the AI reasoning process, other network structures become relevant, enabling eventually the transformation from radiological decision support tools to clinical decision-making aids [28].

### General considerations for AI developments for prostate MRI diagnosis

#### Data science prerequisites

Several issues should be critically assessed for AI and deep learning developments in prostate cancer diagnosis. It is important to check that there are clear technical, radiologic, and/or clinical focuses for AI developments.

##### Scientific rigour

Scientific rigour is necessary for clinical applicability, meaning that descriptions of AI algorithms should be provided to enable judgements on the contextual correctness of the application, supported by appropriate experimental set-ups and analyses. The biases of performance should be rigorously evaluated on training and validation data, but more importantly on independent test data during AI development representative for the target population. The test dataset should exclusively contain unseen data and should ideally include data from other sites/sources to minimise such biases.

##### Ground truth

Experience shows that object detection and classification need plentiful well-annotated data provided by experts, to train CAD and AI systems and validate their performance. High-quality datasets are characterized by combinations of imaging, clinical data, and histopathological data and ideally follow-up to document the ground truth of the presence/absence of significant cancer within the prostate gland. Disease-specific metrics for the quality of ground truth data (e.g. target Gleason score, growth patterns, surface area, volume) need to be accurately established to correctly rate a given algorithm's underlying dataset relative to the natural history of the targeted prostate cancer. These ground truth data are difficult to compile, as digital annotations of prostate MRI scans are rarely performed in clinical practice, and histopathologic correlation with whole gland sampling [30] for MRI verification does not happen in the clinical routine. For example, men with negative scans may not undergo biopsy and men with positive scans may only undergo targeted biopsies.

##### Large datasets

A single deep learning model directly classifying images typically requires a large amount of data for training. For example, over 100,000 expertly annotated retinal images were needed to train a deep learning model that predicted the presence and type of diabetic retinopathy at or above the performance level of clinical experts [31]. The same deep learning algorithm would not achieve the same performance with fewer training cases. Similarly, the performance of deep learning systems for prostate cancer diagnosis is expected to improve when larger datasets are used for training. It is possible that transfer learning methods (e.g. using pretrained models from other image recognition tasks and train them for the task at hand) could improve performance on smaller datasets; however, the total volume of data needed will remain substantial. Similarly, data augmentation, that is the addition of systematically modified (e.g. rotated/skewed/noisy) versions of the images to the training dataset, improves the training process at a technical level. However, data augmentation does not introduce a broader clinical variability which is essential to fully represent the imaging quality characteristics of the data obtained in practise. AI systems will also need to have adaptive properties and be able to use images from a variety of MRI machines, with the wide breadth of pulse sequences and protocol variations often seen in the clinical routine. Demonstration of robustness to such variations or procedures and to retain AI algorithm accuracy will be important for clinical adoption. While many current approaches use deep learning approaches, it is important to note that non-deep learning models (random forests, SVMs, XGBoost) are used in research and commercial applications and can achieve comparable or better performance compared to deep learning until very large datasets are available [32, 33].

#### Study design

##### Segmentation

When evaluating the performance of AI systems, it is paramount to benchmark against the collective performance of general and specialized radiologists as far as possible. These evaluations should be performed at an anatomical level by assessing the quality of prostate gland segmentations at all prostatic levels because radiologists have greater variability when delineating the gland at the prostate apex and base when only axial images are used [34].

##### Outlining

Similarity scores for detected lesions within the prostate gland by AI systems against radiologists tend to be low and dependence of lesion similarity scores with tumour size or aggressiveness measurements needs to be investigated. When considering this, we must acknowledge that the “truth” of lesion outlining by radiologists may also not be accurate as there are no recognized operating procedures on how lesion outlining should be done. Radiologist outlining is often erroneous when verified against whole-mount histopathology [35] but is better for index prostatic lesions when verified against step-section mapping biopsies [1]. Bringing objectivity to the lesion outlining process for AI training would require imaging data with histopathologic correlations from either prostatectomy specimens or transperineal template mapping biopsies. Ideally, the under-sampling and sampling errors of targeted biopsy alone or in combination with systematic biopsy should be taken into account for AI detection and lesion outlining tasks.

##### bpMRI

To date, most AI developments have utilized bpMRI for system development, often using axial plane imaging only for training and validation. bpMRI could help to more efficiently manage scarce MRI resources for broader availability of prostate MRI where needed [36]. However, both dynamic contrast enhancement (DCE) and multiplane imaging assessments are part of the PI-RADS standard and are mandatory for other systems that evaluate prostate MRI for the presence of significant cancers [37, 38]. It is also important to remember that while multiparametric, multiplane MRI has been extensively clinically validated, bpMRI with single axial imaging plane imaging has not undergone such rigorous evaluations for being able to direct prostate biopsies [14]. With promising studies underway to ascertain the value of bpMRI alone [39], this might change in the future. Readers should note that in the sub-analysis of the 4M data there was a 70% increase in the uncertain category when single plane bpMRI was evaluated [12]. Although the overall radiologic diagnostic performance differs between bpMRI and mpMRI is likely to be small for experienced readers working in multiple imaging planes [12], we need to be cognizant of the potential for error propagation introduced by axial plane bpMRI evaluations which could mean that the desired biopsy impacts may not be realized because of more uncertain cases. Nevertheless, the way DCE imaging is performed across institutions also varies considerably in terms of sequence/acquisition parameters and bolus timing, thus adding another challenge to the standardization task for tools based on image analysis and their reproducibility [40–42].

##### Radiological decision support tool

Remembering that the PI-RADS score has been validated for clinical biopsy decision-making, we would caution deep learning developments that predict PI-RADS category scores based on bpMRI sequences. Thus, any deep learning tool that actively promotes axial-only bpMRI approaches, seeking indirect validation via the use of mpMRI PI-RADS scoring system, needs further validation.

These considerations point to the important need for AI systems to be developed initially as radiological decision support tools rather than clinical decision-making aids. A change in indication from a “radiological diagnosis support tool” to “clinical decision-making tool” would need to undergo rigorous testing against clinically valid endpoints, such as the presence of clinically significant lesions in positive and negative cases.

##### Data variability and validation

Each AI system developed for prostate cancer diagnosis will require testing in prospective, multivendor, multi-institutional studies to assess system performance, from which robust measurements of clinical impacts can be derived in different use case scenarios as discussed in more detail below. Currently, very few studies involving AI in medical imaging are conducted in a standardized and comparable way. A recent analysis of over 31,000 studies carried out from 2012 to 2019 could only identify 82 studies which allowed a meaningful comparison and had end-to-end validation with medical professionals [43]. Therefore, in the phases of optimization of AI systems, larger, well-curated, diverse (potentially from multiple vendors, multiple centres) training and validation datasets must be developed, with spatially correlated histopathology validation, and all studies need to be rigorously tested against human performance by multiple readers. The single institution/single vendor ProstateX dataset is an important step in this direction [44], and the concept of hidden test data could be used to provide means for objectively estimating a network’s performance against an alternate algorithm.

Advanced assessments of the performance of AI systems should seek to go beyond technical evaluations of similarity coefficients, receiver operator curve, and precision-recall curves assessments which may be enough for “radiological diagnosis support tool” developments. However, “clinical decision-making tool” developments will require additional assessments of potential clinical impacts which should be incorporated into assessments of performance as early as possible, including net-benefit analyses, thus accelerating general clinical acceptance.

#### Patient selection

##### Cancer prevalence—pre-test probability

It is important to remember that the documented benefits of MRI only emanate from western populations presenting for secondary screening, where the prevalence of the International Society of Urological Pathology (ISUP) prostate cancer grades 2–5 cancer is about 30–50% in routine clinical practise. Since most AI systems are trained on these datasets, their performance is “tuned” for this prevalence of the disease. We cannot therefore directly extrapolate AI performance to men with a much higher or much lower prevalence of significant cancers or for other histologic csPCa definitions. AI systems will need to adapt to the disease prevalence of the local population and to have performance characteristics (acceptable false-positive and false-negative rates) that enable the delivery of diagnostic benefits according to clinical priorities at differing disease prevalence (see below).

#### Patient selection

In this regard, it is especially important to note that currently available MRI dataset developments exclude multiple patient groups. However, the entire population presenting for diagnosis must be represented in the training/validation datasets for general prostate cancer diagnosis AI developments. Differences between populations need to be recognized, such as the prevalence for reasons discussed above and due to different anatomical locations of significant cancers within population groups [34]. Therefore, in addition to AI algorithms for biopsy-naïve and prior negative biopsy men, other patient groups should also be included, such as men with prior negative or equivocal MRI scans who do not undergo an immediate biopsy on safety net follow-up regimens and patients under active surveillance for known low-risk prostate cancer. MRI datasets may be expanded to patients with treated diseases in due course. Patient groups excluded in AI developments should be “black-boxed” for the indication (carry serious safety risks).

#### AI developments as radiological diagnosis support tools

##### Differences in tolerance to false results

As deep learning–based MRI interpretations will assist in determining the need for a biopsy after radiological interpretations, the clinical setting will determine the appropriate level of tolerance to false-positive or false-negative results. Clinical tolerance to false-positive results differs between patient groups [7]. In biopsy-naïve men, there is a need to minimize over-testing and detection of indolent cancers and to detect significant cancers likely to cause harms; the former consideration, therefore, reduces the acceptability of false-positive results. In the same vein, men undergoing primary screening for prostate cancer at a low background risk of significant cancer have general intolerance to false-positive results because of the harms related to over-testing. However, there also needs to be a low false-negative rate to aggressive disease. On the other hand, in men with persistent suspicion after negative findings on a previous biopsy, it is essential not to miss potentially aggressive cancers; therefore, the tolerance to false-positive results is higher. These tolerances need to be factored into AI design parameters.

##### High false positives

A high false-positive rate of AI systems has been noted by multiple investigators [18, 19, 45, 46]. For example, Schelb et al [45] noted that the success of their AI system developed on bpMRI came at the cost of a high false-positive rate of around 50%. The AI system’s sensitivity rapidly declined when attempting to lower the false-positive rate. That is, to maintain high sensitivity for the detection of clinically significant cancer, the AI system overcalled multiple lesions that did not represent significant cancers (false-positive findings).

##### Performance measures

Discrimination and calibration are important indicators for evaluating the performance of risk prediction or decision tools [47]. While discrimination focuses on separating people with disease from people without disease, calibration focuses on the agreement between observed outcome and predicted risk.

Discrimination is important for AI developments in the prostate cancer diagnostic settings, because we want to separate men with from men without clinically significant prostate cancer. Good discrimination means that men with significant cancer will consistently have higher predicted risks than those men without significant disease. To indicate the discriminative ability of risk prediction models for a binary outcome, the area under the receiver operating characteristic (ROC) curve is commonly used, which plots the sensitivity (true-positive rate) against 1 specificity (false-positive rate) for consecutive cut-offs for the probability of significant disease. In general, discrimination is not dependent on disease prevalence.

Calibration refers to the agreement between observed outcomes and predictions. Calibration is more important in prognostic settings, because we would like to more precisely predict the risk of clinically significant prostate cancer. Calibration concerns itself directly with the estimated probabilities or predictive values. The positive predictive value is defined as the probability of clinically significant disease given a positive test result, and the negative predictive value is the probability of no significant disease given a negative test result. When a risk score is used, the continuous analog is the probability of disease (i.e. prevalence) given the value or range of the score. An assessment of calibration directly compares the observed and predicted probabilities, for which the disease prevalence is very important.

#### AI developments for clinical decision-making

Multiple tasks can be envisioned for AI developments to meet clinical needs. These tasks should not be considered as distinct AI developments but rather as desired integrations that will enable the successful clinical development as “clinical decision-making tools” helping to deliver clinically meaningful benefits in men with suspected prostate cancer.

##### AI in low-risk men

For biopsy-naïve men, AI systems should initially focus on identifying men with normal/non-suspicious findings with low false-negative detection rates (high sensitivity) at a certain level of specificity, so that further testing can be avoided. If we accept that the false-negative rate of negative MRI is < 10% [48], it is estimated that about 1 in 3 biopsy-naïve men and 2 in 3–5 men after prior negative biopsy [1, 49] can avoid biopsies after a negative MRI with a low clinical suspicion when evaluated by expert human readers. The performance level for AI in this regard will need to be firmly established.

##### AI in high-risk men

**Fig. 2:**
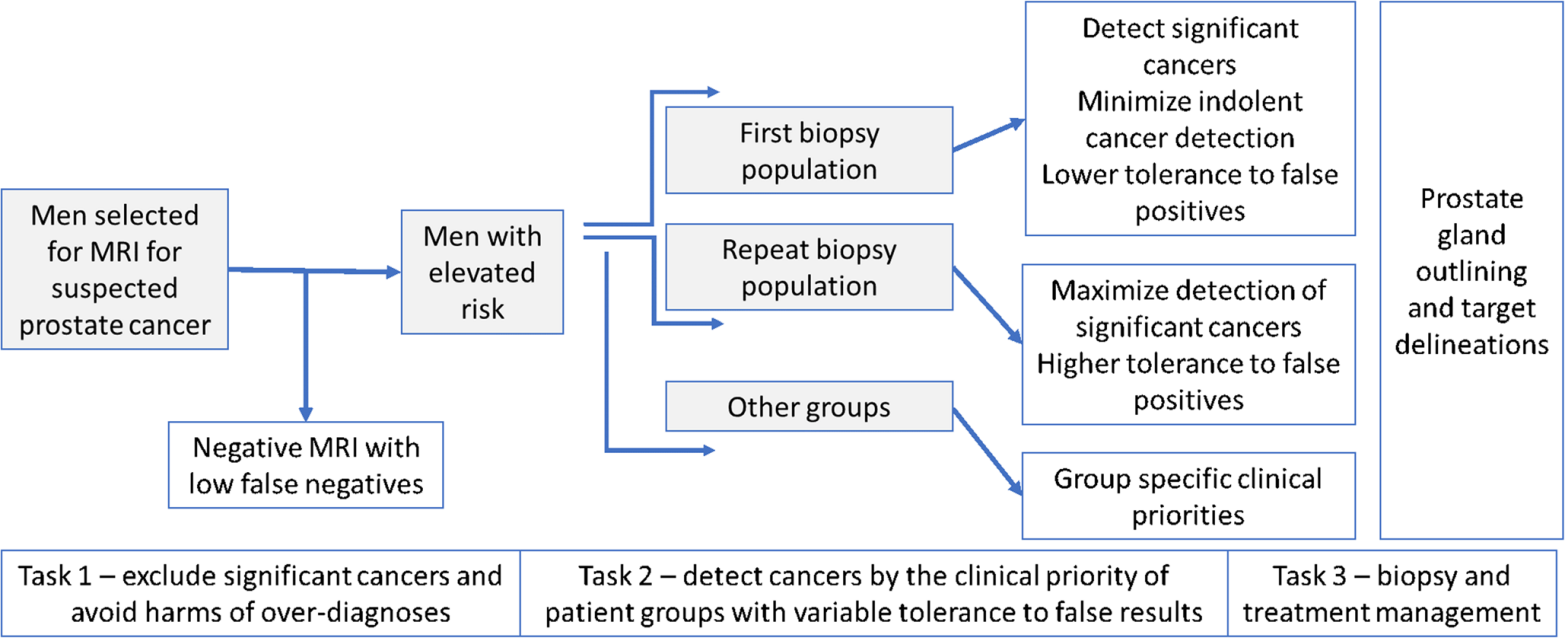
Developing AI systems as clinical decision-making tools: Stepwise application of AI according to population characteristics can help deliver clinically appropriate benefits by considering clinical risk profiles and clinical priorities. Multiple tasks can be envisioned that are directly relevant to the planning of biopsy and treatment tasks

Men with a higher level of suspicion will have a higher prevalence of clinically significant cases and will require accurate lesion identification and outlining according to patient clinical care priorities (Fig. 2). The results from the detection workflows will need to be directly relevant to the subsequent planning of biopsy and treatment tasks.

##### AI as a triage tool

In the short term, the disadvantage of high false-positive rates can be addressed by using AI systems as triage tools that detect and present suspicious lesions, along with their delineations to radiologists. In a second step, radiologists can either accept or reject proposed lesions, by considering clinical risk factors and patient priorities and improve delineations of cancer-suspicious targets before proceeding to reporting and communication tasks. The latter activities are radiological diagnosis support tool developments. Schelb et al [45] noted that when radiologists and machine learning agree on the likely presence of clinically significant lesions, the positive predictive value increased without affecting the negative predictive value. However, it should be noted that the lesions brought into the attention of radiologists will still need to be evaluated by radiologists and the characteristics of the learning curve for an ideal interaction between the AI and radiologists remains unknown. Naturally, it would be important to understand why there are higher false-positive results with AI systems compared to experienced radiologists.

### User interface considerations

#### AI and radiologist interactivity

Using interactivity, suspicious lesions should be detected, delineated, graded for the likelihood of malignancy, and if possible given probabilities of aggressiveness. Detected lesions could be classified according to their PI-RADS category scores thus enabling image-based risk-stratification when presented with PI-RADS compliant multiplane mpMRI scans. Delineation of cancer-suspicious lesions with high similarity to actual tumour locations/sextants/delineations will assist in planning biopsy and focal therapies and boost radiation therapy indications. The additional information from the interaction of the radiologist with the AI results—i.e. accepting and rejecting lesions suggested by deployed algorithms—could then be used to further improve the AI networks and/or be used to give feedback to the readers.

#### AI supporting radiologist’s workflow

Furthermore, AI-based reading systems should be incorporated into user interfaces for the general reading of prostate MRI and not—as previously seen in many CAD systems—offer static captures of image analyses. User interfaces should be tailored to the radiologists’ workflow allowing for the comprehensive evaluation of MRI along with the results of the AI subsystem. Additional capabilities should include automatic structured reporting according to and in addition to the PI-RADS standard as appropriate.

An immediate use case of AI and computer-aided detection systems derives from the high performance of AI algorithms in segmenting the entire gland and prostate zonal anatomy comparable to manual segmentations. This opens the possibility for segmentation of the prostate gland to be used for cognitive and fusion-biopsy as well as for radiation therapy planning. In the first instance, AI systems should detect normality with high confidence. This triage role would have an immediate benefit for radiological workload and help build the confidence of AI capabilities.

## Image acquisition

Multiple additional applications of AI systems in MRI prostate cancer diagnosis can be envisioned. These include real-time monitoring of MRI data acquisitions and the interactive deployment of remedial actions while scanning is ongoing, in order to deliver optimized diagnostic images for every examination. This would enable consistency in the planning and acquisition of MRI exams and improve the comparability of subsequent scans. It is important to remember that AI algorithms work best when data acquisitions are consistent. Another in-scanner application could be for deciding which patients need contrast medium injection where bpMRI is performed initially, which would help increase efficiency and reduce costs if contrast medium is not deployed, or to reduce indeterminate cases when contrast medium is used in appropriate bpMRI cases.

### Image quality

A helpful AI system should be able to evaluate the prostate gland even when MR images are not ideal but should also indicate when quality is insufficient for the specific AI algorithm to work effectively. As already noted, the focus of AI should initially be on the detection of men with a low likelihood of having clinically significant cancers and thereafter the detection, classification, and delineation of suspected cancer locations in men not deemed to be negative at high negative predictive value. This will help in optimizing reading workflows of increasingly complex mpMRI data and help ameliorate radiological demands.

### Follow-up imaging or second reading

Another aspect, currently omitted from AI developments, should be the consideration of multiple imaging time points when training AI systems, as temporal changes provide valuable information for analyses. The latter is especially important for men on PSA surveillance who have avoided immediate biopsy after negative MRI as part of a safety-net regimen of follow-up. Similar capabilities would also be of value for men undergoing active surveillance. A role as a second reader for indeterminate MRI cases that includes integrations with clinical history, biochemistry, and genomic profiles can also be envisioned.

### Personalized diagnosis

**Fig. 3:**
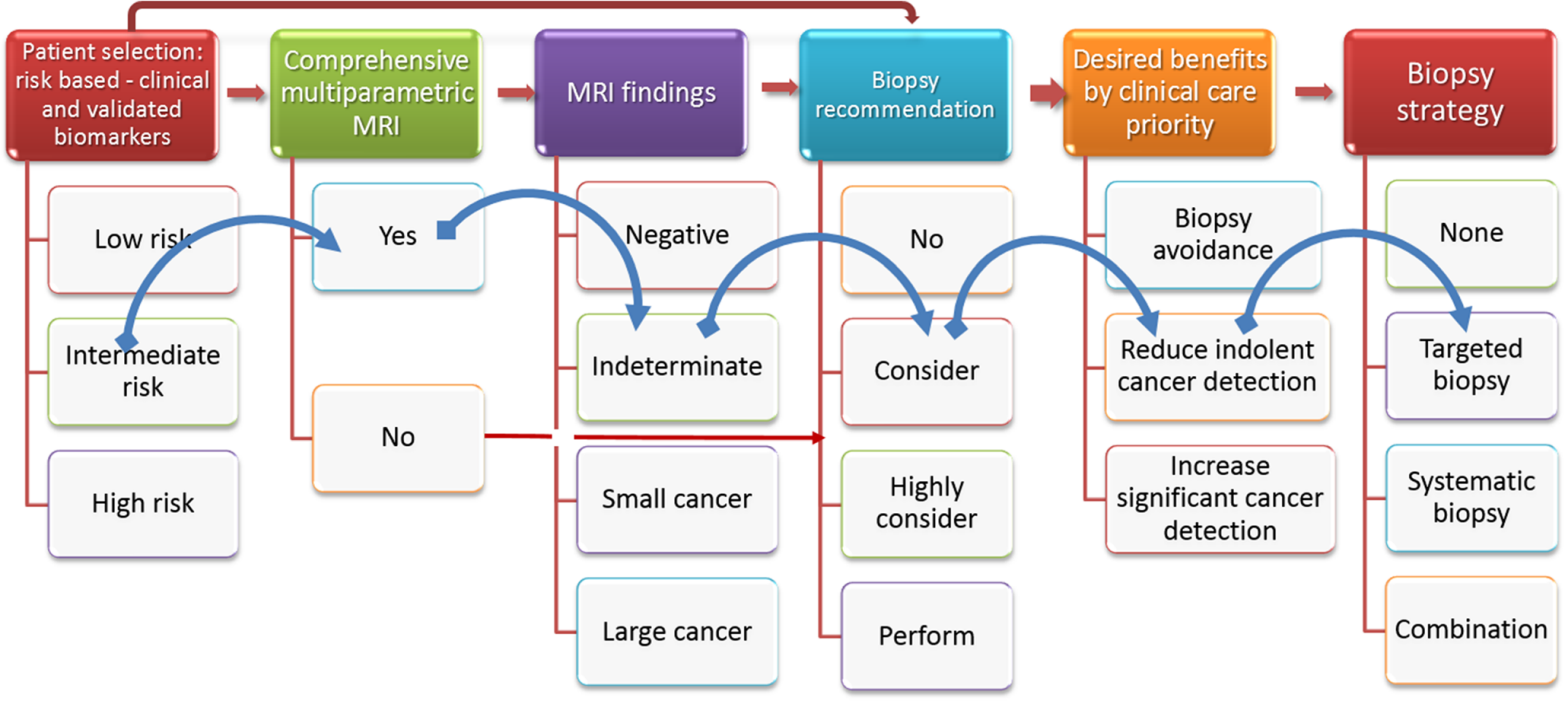
Personalizing diagnosis of prostate cancer using validated decision support tools: Reimagining prostate cancer diagnosis requires validated AI decision support tools that integrate imaging and blood biomarkers to delivery personalized diagnoses via patient selections and biopsy management. The blue arrows point to a typical man with an elevated risk of prostate cancer. In the first step, there needs to be a decision on the need for a comprehensive multiparametric approach as opposed to a simpler biparametric approach. If there is an indeterminate MRI result, the need for biopsy will require integration with clinical risk factors and his clinical care priorities. For a man seeking to minimise over-testing, a targeted biopsy alone can be envisioned. Several other clinical scenarios can be similarly thought of. High-quality, end-to-end multidisciplinary working of the diagnostic chain supported by AI systems will be required to deliver this personalized vision of prostate cancer diagnosis

AI systems can enable the combination of MRI and multivariate risk prediction tools to personalized prostate cancer diagnoses. For example, while multivariate risk-based models can be used to decide on the need for MRI, AI enables the combination of MRI results and risk-based models for biopsy decisions, and in so doing it helps to tailor biopsy strategies in order to deliver desired personalized patient care benefits (Fig. 3). This is because each biopsy approach will have trade-offs of benefits and harms, based on the desired benefits of improved diagnostic yields, reduced biopsy testing, or reduced detection of indolent prostate cancer. AI tools can, therefore, support physicians and patients in increasingly complex biopsy decisions with the advent of serum, urinary, and imaging biomarkers.

## Conclusion

The ESUR Prostate MRI Working Group and the ESUI recognise that further developments in artificial intelligence (AI) are essential to successfully deploy community-wide MRI-driven prostate cancer diagnosis. AI system developments should ensure that the main benefits of biopsy avoidance are delivered while decreasing the variations in biopsy yields according to MRI suspicion levels. This white paper identified several domains in AI developments and pointed out an envisioned direction of travel. Since all current AI systems for prostate cancer detection are experimental, multiple developmental efforts are still needed to bring the vision to fruition for all men with suspected prostate cancer. Initial work should focus on AI developments to support radiologists’ workflow including gland and target outlining tasks for fusion biopsies. Developing AI systems as clinical decision-making tools will require greater efforts. The latter encompass larger multicentric, multivendor datasets where the different needs of patients stratified by diagnostic settings, disease prevalence, patient preference, and clinical setting are considered. Standard operating procedures will increase the confidence of patients and payers, thus enabling the wider adoption of the MRI-directed approach for prostate cancer diagnosis.

## Abbreviations

ADC: Apparent diffusion coefficient
AI: Artificial intelligence
bpMRI: Biparametric MRI
csPCA: Clinically significant prostate cancer
DCE-MRI: Dynamic contrast-enhanced MRI
mpMRI: Multiparametric MRI
MRI: Magnetic resonance imaging
PI-RADS: Prostate Imaging – Reporting and Data System

## Declarations

### Guarantor

The scientific guarantor of this publication is Dr. Padhani.

### Statistics and biometry

No complex statistical methods were necessary for this paper.

### Methodology

• Not applicable
